# Understanding factors influencing the adoption of moxibustion techniques by the population: an extended study based on the UTAUT model

**DOI:** 10.3389/fpubh.2025.1508716

**Published:** 2025-05-21

**Authors:** Chengxin Fan, Zina Fan, Qiusha Li, Zixuan Zhao, Chunxiao Yang, Zhongming Chen, Wenqiang Yin

**Affiliations:** ^1^School of Public Health, Shandong Second Medical University, Weifang, China; ^2^“Health Shandong” Severe Social Risk Prevention and Management Synergy Innovation Center, Weifang, China; ^3^School of Public Health, Tongji Medical College, Huazhong University of Science and Technology, Wuhan, China; ^4^School of Law, Linyi University, Linyi, China; ^5^School of Management, Shandong Second Medical University, Weifang, China

**Keywords:** moxibustion, e-UTAUT, perceive risk, SEM, multi-group analysis

## Abstract

**Background:**

Traditional Chinese medicine plays a unique role and has proven efficacy in preventing and treating common and chronic diseases. Moxibustion, as a type of traditional Chinese medicine healthcare therapy, has a broad mass, social, and cultural foundation in China. This study analyzes the pathways and influencing factors of residents' acceptance of moxibustion.

**Methods:**

Data were collected from 808 residents in 18 cities or districts using whole cluster stratified random sampling. Take the expanded Unified Theory of Acceptance and Use of Technology model scale as the research tool. Data were analyzed by SPSS 25.0 and AMOS 24.0, including descriptive statistics, one-way analysis of variance, structural equation model analysis, and multi-group model analysis.

**Results:**

Structural equation modeling showed that performance expectancy (β = 0.603, *p* < 0.001), effort expectancy (β = 0.260, *p* < 0.001), social influence (β = 0.373, *p* < 0.001), and perceived risk (β = −0.162, *p* < 0.001) significantly predicted behavioral intention. Facilitating conditions (β = 0.186, *p* < 0.01) and behavioral intention (β = 0.708, *p* < 0.001) directly affect usage behavior. The multiple-group analysis found that experiential and chronic disease status played a moderating role in the structural pathways.

**Conclusions:**

The study confirmed that the constructed resident moxibustion technology model can serve as a suitable framework for predicting the factors that influence residents' intention to use moxibustion and their behaviors. Increasing residents' performance expectations and effort expectations, creating a positive social environment, and reducing perceived risk are key factors in enhancing residents' behavior and willingness to use moxibustion.

## 1 Introduction

Traditional Chinese medicine (TCM) has long been integral to China's healthcare system, valued for its rich historical legacy and distinctive therapeutic techniques. In recent decades, TCM has gained global relevance as a cost-effective and culturally accepted form of complementary therapy. This expanded role reflects not only its historical importance but also its potential to address modern public health challenges through preventive care and integrative treatment strategies. Approximately 60% of the population in Hong Kong or Mainland China have consulted TCM practitioners at least once, and 60%−75% of the population in Taiwan, Japan, Korea, and Singapore use traditional medicine at least once a year (1). The use of TCM is also widespread in other parts of the world (2). In the United States, TCM is classified within complementary and alternative medicine (CAM). According to the 2022 National Health Interview Survey (NHIS), the percentage of adults using CAM increased from 19.2% in 2002 to 36.7% in 2022 (3). In Australia, a robust and comprehensive legislative and regulatory framework governing Chinese medicine has been established (4). The nation currently boasts over 4,800 registered practitioners and maintains an extensive educational infrastructure dedicated to Chinese medicine (5, 6). Extensive research has demonstrated TCM's efficacy in managing chronic conditions such as cancer, diabetes, cardiovascular disease, epilepsy, Alzheimer's disease, Parkinson's disease, depression, and cerebral ischemia (7–11). In addition, TCM has demonstrated its key role in dealing with SARS and the novel coronavirus (COVID-19) (12, 13). These trends underscore TCM's expanding global appeal and emphasize the need to investigate the determinants of its acceptance at the interface between traditional practice and modern health promotion.

Moxibustion, a type of TCM therapy, has been proven to improve blood circulation and promote overall health. Clinically, it is applied to diverse conditions with demonstrated efficacy. Its simplicity and low cost cater to the needs of residents, while its thermal application suits various therapeutic indications. Moxibustion mainly treats by burning Chinese medicinal materials, producing thermal stimulation that primarily achieves warming and tonifying effects (14). After thousands of years of inheritance and development, moxibustion enjoys a broad popular, social, and cultural foundation in China. Its simplicity and cost-effectiveness confer unique advantages in addressing patient healthcare needs (15, 16). According to the Yearbook of traditional Chinese medicine of China, moxibustion represents 12.8% of all TCM technique usage (17). In Taiwan, 14.9% of adults reported moxibustion use in the past year (18). Historical analyses indicate that moxibustion has been applied mainly to pain, inflammatory bowel disease, hip disorders, irritable bowel syndrome, osteoarthritis, stroke, and inflammation (19). These predominantly chronic conditions suggest that moxibustion aligns well with contemporary preventive healthcare needs.

Moxibustion's significance in healthcare and disease prevention is well-established. However, most research has concentrated on clinical trial applications (20, 21), technology innovation (22, 23), and safety assessments of moxibustion (24, 25), emphasizing its technical utility. At the same time, moxibustion is a treatment that utilizes the thermal effect produced by burning moxa floss, which inevitably produces burning or smoke irritation during the treatment process, resulting in a certain perceived risk to the patient when using it. Historical studies have found that moxibustion therapy can also pose certain safety concerns, including potential tissue damage and adverse physical reactions, such as burns (26), scalds (27), and smoke pollution (28). Consequently, perceived safety and efficacy uncertainties may influence individuals' willingness to pursue moxibustion. There have been fewer investigations into residents' willingness to accept moxibustion techniques and their behaviors, and little literature to explore the mechanisms behind residents' behaviors. Therefore, understanding the factors that influence residents' adoption and continued use of moxibustion is essential for enhancing the overall health of the community.

In this study, we extend the Unified Theory of Acceptance and Use of Technology (UTAUT) by integrating perceived risk constructs specific to moxibustion to develop a comprehensive acceptance model. We operationalized the model through a structured questionnaire to assess both acceptance intentions and usage behaviors. The findings aim to inform the optimization and development of moxibustion and related TCM service delivery. In this study, we attempt to address the following two questions:

Research Question 1: What are the influencing factors on residents' willingness and behavior to use moxibustion?

Research Question 2: Can the constructed resident moxibustion technology model have good predictive ability for residents' willingness and behavior to use moxibustion?

## 2 Theoretical framework and hypotheses

### 2.1 Theoretical background

The UTAUT model is a comprehensive theoretical model proposed by Venkatesh et al. It synthesizes commonly used theories in the field of technology acceptance research. Empirical studies have demonstrated the UTAUT model's effectiveness in examining individuals' intentions and behaviors regarding technology adoption, including the influencing factors and interrelationships among variables (29). Previous studies have shown good applicability in applying the UTAUT model to the field of healthcare technology (30). Utilizing the UTAUT model to explore the factors influencing residents' acceptance of moxibustion can uncover challenges and limitations in promoting TCM such as moxibustion.

**Figure 1:**
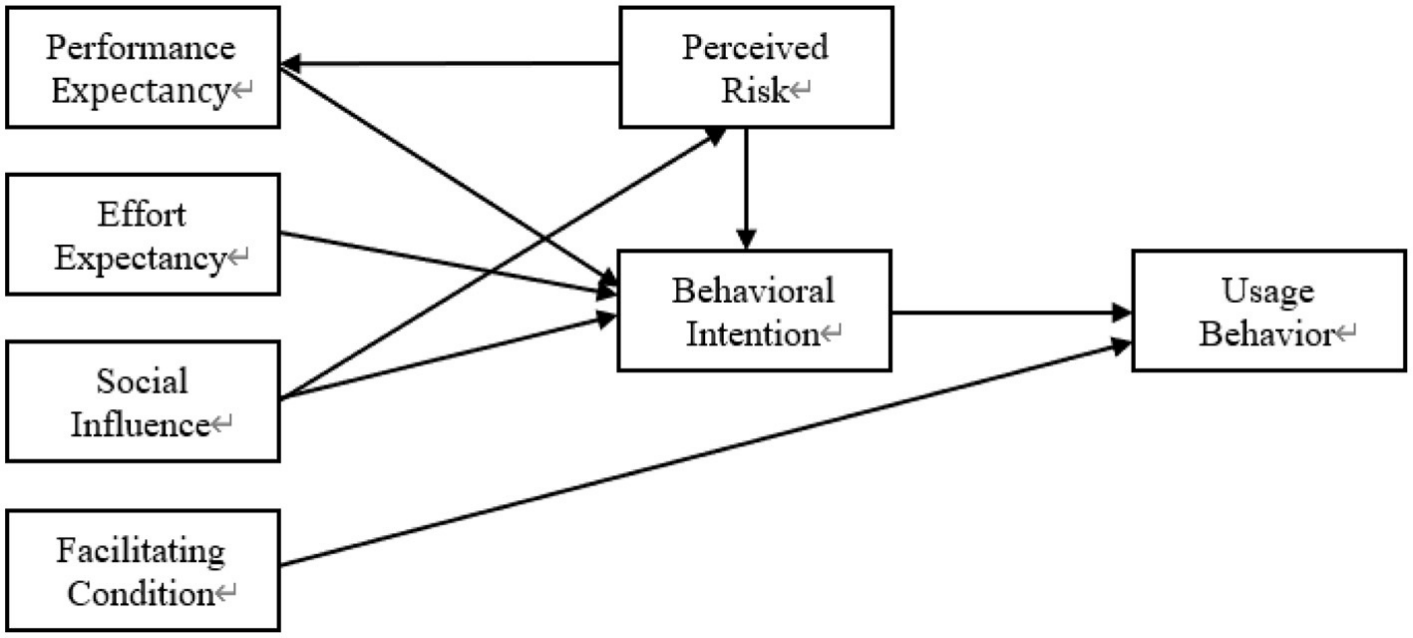
Moxibustion acceptance model.

This study uses the UTAUT model as the theoretical framework for the research. Considering potential risks associated with moxibustion, this study extends the UTAUT model by integrating perceived risk as an additional construct. We modeled moxibustion acceptance using seven dimensions (Figure 1): performance expectancy (PE), effort expectations (EE), social influence (SI), facilitating conditions (FC), perceived risk (PR), behavioral intention (BI), and usage behavior (UB). In order to ensure the validity and applicability of the measurement indices, most of the relevant measurement items in this study were derived from previous related studies and optimized according to the characteristics of moxibustion techniques. Subsequently, the measurement items are operationalized based on the defined constructs, tailored to the specific nature of the research problem and the focus of this paper (Table 1).

**Table 1:** Items and their sources.

**Construct**	**Corresponding items**	**Items sources**
Performance expectancy (PE)	PE1. I think moxibustion helps to strengthen the body	(29, 31)
	PE2. I think moxibustion can cure diseases	
	PE3. I think moxibustion has the same effect as injections and pills	
Effort expectancy (EE)	EE1. Learning how to use moxibustion is easy for me	(29, 32)
	EE2. Learning moxibustion techniques and related contraindications was easy for me	
	EE3. I find moxibustion service easy to use	
Social influence (SI)	SI1. People who are important to me think that I should use moxibustion	(29, 33, 34)
	SI2. People who influence my behavior think that I should use moxibustion	
	SI3. People who are medical professionals think that I should use moxibustion	
Facilitating condition (FC)	FC1. There is a moxibustion facility nearby for people to receive moxibustion	(29, 35)
	FC2. There are professionals for convenient consultation in the use of moxibustion problems	
	FC3. There is a facility for buying moxibustion equipment and materials	
Perceived risk (PR)	PR1. I'm concerned that moxibustion isn't scientifically sound	(36, 37)
	PR2. I'm concerned about the health risks of moxibustion fumes to myself and others	
	PR3. I'm concerned about the health risks of using moxibustion (burns, scalds)	
Behavioral intention (BI)	BI1. I am willing to use moxibustion	(29, 38, 39)
	BI2. I am willing to recommend moxibustion to others	
	BI3. I hope to continue using moxibustion in the future	
Usage behavior (UB)	UB1. I often learn about moxibustion and related knowledge	(29)
	UB2. I will recommend moxibustion to people around me	
	UB3. I will continue using moxibustion in the future	

### 2.2 Performance expectancy (PE)

Performance expectancy refers to “the degree to which an individual believes that using the system will help him or her to attain gains in job performance” (29). In this study, PE refers to the residents' belief that the use of moxibustion techniques can cure their illnesses, strengthen their bodies, and have a complementary effect compared to other healing modalities. Venkatesh et al. found that PE is the strongest determinant of users' BI to adopt technology. Meanwhile, some scholars found that PE has a direct positive effect on BI (31). The above discussion led this study to posit the following hypothesis:

H1: PE has a positive impact on residents' intention to use moxibustion.

### 2.3 Effort expectancy (EE)

Effort expectancy refers to “the degree of ease associated with the use of the system” (29). In this study, EE pertains to residents' perceptions regarding the ease of using moxibustion techniques, acquiring related knowledge, and integrating these practices into their daily routines. Previous research indicates that the complexity and specificity of moxibustion may hinder its adoption among residents. Notably, Seethamraju et al. (32) found that EE has a significant impact on users' willingness to adopt health information systems. Hence, it was hypothesized that:

H2: EE has a positive impact on residents' intention to use moxibustion.

### 2.4 Social influence (SI)

Social Influence refers to “the degree to which an individual perceives that important others believe he or she should use the new system” (29). Li and Zhao (33) have found that SI significantly impacts users' intentions to adopt technologies. According to the Health Behavior Model, SI serves as a catalyst influencing residents' attitudes toward technology adoption. When individuals perceive that significant others hold favorable views toward moxibustion, their own intention to engage with the practice is likely to increase (34). Therefore, this study proposes the following research hypotheses:

H3: SI has a negative impact on residents' perceived risk to use moxibustion.
H4: SI has a positive impact on residents' intention to use moxibustion.

### 2.5 Facilitating condition (FC)

Facilitating condition refers to “the degree to which an individual believes that an organizational and technical infrastructure exists to support the use of the system” (27). Tomić et al. (35) found that FC as a direct determinant influencing both behavioral intention and actual technology usage. In the context of TCM, moxibustion necessitates specific resources and infrastructure to support its practice and dissemination. The availability and accessibility of such resources can enhance residents' motivation to adopt and continue using moxibustion. Therefore, this study hypothesizes:

H5: FC has a positive impact on the actual use of moxibustion.

### 2.6 Perceived risk (PR)

Perceived risk refers to “the degree of uncertainty of individuals regarding the expected outcomes of using technology” (36). In this study, PR pertains to the extent of skepticism residents harbor toward moxibustion. Irimia et al. (37) found that PR has a negative impact on user willingness. Given that medical technologies are intrinsically linked to the immediate needs of the population, individuals tend to exhibit heightened sensitivity toward their safety. Consequently, residents' perceived risk associated with moxibustion can directly influence their perceived ease of use, subsequently affecting their willingness to adopt the practice. Hence, this study proposes the following research hypotheses:

H6: PR has a negative effect on residents' performance expectations.
H7: PR has a negative effect on residents' intention to use moxibustion.

### 2.7 Behavioral intention (BI) and usage behavior (UB)

Behavioral intention refers to “the degree of an individual's subjective intention to use a technology or service” (29). Extensive research across various domains has established a strong association between BI and UB, indicating that BI serves as a reliable predictor of UB (38, 39). Wang et al. (40) demonstrated that BI significantly forecasts the actual utilization of health information technologies. Based on the above literature, this study posited the following hypothesis:

H8: BI has a positive impact on the actual use of moxibustion.

## 3 Methods

### 3.1 Measurement instruments and questionnaire design

A structured questionnaire was developed initially in English and then translated into Chinese by two independent experts whose native language is Chinese, both of whom possess extensive experience in health management and are well-versed in the theoretical underpinnings of moxibustion and UTAUT modeling. The questionnaire comprises two parts. The first part collects demographic information, including age, gender, education level, personal annual income (Yuan), moxibustion usage experience, and health status. The second part assesses the seven key constructs of the Acceptance Model of Moxibustion Techniques—Performance Expectancy, Effort Expectancy, Social Influence, Facilitating Conditions, Perceived Risk, Behavioral Intention, and Usage Behavior, each measured by three items, totaling 21 items. These items were adapted from established, validated scales and refined through a pilot test to ensure clarity and contextual appropriateness. All items are rated on a 5-point Likert scale (1 = “strongly disagree” to 5 = “strongly agree”).

### 3.2 Data collection

In this study, a survey was conducted from November 2020 to January 2021 in Weifang City, Shandong province. Stratified random sampling was used to select the participants. Three counties (cities or districts) were randomly selected from Weifang, based on the population size and the level of economic and social development, categorized as high, medium, and low. Three township health centers (rural areas) and three community health service centers (urban areas) were randomly selected in each county (city or district) based on the proportion of permanent residents in each area. On the basis of the selected primary medical institutions, residents are randomly selected using the public health platform. Eligible participants were adults aged 18 years or older, registered residents at selected health centers, who were cognitively and physically able to complete a written survey. Individuals with severe hearing, speech, or cognitive impairments were excluded. A total of 850 questionnaires were distributed in this study and 809 valid questionnaires were recovered, with a valid recovery rate of 95.18%. Previous research evidence proves that the sample size should be at least 10 times the number of items in the study (41, 42). Given the 21 items in this study, the sample size was deemed adequate.

### 3.3 Data analysis

In this study, IBM SPSS 25.0 was used for descriptive and exploratory analyses and AMOS 24.0 was used for structural equation model (SEM) analysis. Statistical significance was set at *p* < 0.05. First, frequencies (*n*) and percentages (%) were calculated to describe the basic demographic characteristics of the participants. Second, mean values (*x*) and standard deviations (SD) were computed for each construct within the moxibustion acceptance model. One-way analysis of variance (ANOVA) was performed to assess differences across groups. Reliability and validity analyses were conducted to evaluate the measurement model. Lastly, SEM was used to test the hypothesized behavioral pathways. To adjust for multiple hypothesis testing, we applied a Bonferroni correction, setting the adjusted α at 0.00625 (0.05/8) for each of the eight tests. Model fit was evaluated using the following indices: Root Mean Square Error of Approximation (RMSEA), Goodness-of-Fit Index (GFI), Comparative Fit Index (CFI), Incremental Fit Index (IFI), Normed Fit Index (NFI), and Tucker-Lewis Index (TLI). Multi-group analyses were conducted separately for each key demographic characteristic to prevent multicollinearity among covariates and to clearly highlight differences between subgroups.

## 4 Results

### 4.1 Demographic characteristics of sample

The demographic characteristics of the sample are presented in Table 2. Among the 809 participants, 63.66% were males, and the majority of respondents (45.98%) were aged between 46 and 69 years. In terms of educational qualifications, 61.68% of the participants had achieved at least a junior high school level of education. About 51.79% of respondents had experience using moxibustion.

**Table 2:** Description of socio-demographic characteristics.

**Variables**	**Description**	***n***	**%**
Gender	Male	294	36.34
	Female	515	63.66
Age	18–45	69	8.53
	46–69	372	45.98
	70 or above	368	45.49
Personal annual income (Yuan)	≤ 5,000	353	43.63
	5,001–20,000	233	28.80
	≥20,001	223	27.56
Education qualification	Primary school and below	310	38.32
	Junior high school	266	32.88
	High school or above	233	28.80
Moxibustion usage experience	Yes	390	48.21
	No	419	51.79
Chronic disease	Yes	218	26.95
	No	591	73.05

n, number of participants.

### 4.2 Analysis of residents with different characteristics in each dimension

The mean scores of performance expectancy, effort expectancy, social influence, facilitating condition, perceived risk, behavioral intention, and usage behavior for the participants were respectively 3.68 ± 0.77, 3.50 ± 0.96, 3.96 ± 0.85, 4.07 ± 0.75, 2.30 ± 0.88, 3.73 ± 1.02, and 2.66 ± 1.28. There is a significant difference in usage behavior scores among residents of different age and income groups (*p* < 0.05). The 18–45 age group has the highest average score, and the scores of residents with higher annual income were also higher than those with lower annual income. There is a significant difference in behavioral intention scores among residents with different educational qualifications and usage experiences groups (*p* < 0.05). It showed that residents with high school and above education subgroup had the highest mean scores, and residents with chronic diseases who had used moxibustion had significantly higher mean scores (Table 3).

**Table 3:** Analysis of different characteristics (*M*, SD).

**Variables**	**PE**	**EE**	**SI**	**FC**	**PR**	**BI**	**UB**
Total	3.68 ± 0.77	3.50 ± 0.96	3.96 ± 0.85	4.07 ± 0.75	2.30 ± 0.88	3.73 ± 1.02	2.66 ± 1.28
**Gender**							
Male	3.66 ± 0.76	3.47 ± 1.00	3.95 ± 0.87	4.04 ± 0.79	2.34 ± 0.93	3.70 ± 1.02	2.58 ± 1.30
Female	3.69 ± 0.78	3.52 ± 0.94	3.96 ± 0.83	4.09 ± 0.73	2.28 ± 0.85	3.74 ± 1.01	2.71 ± 1.27
*F*-value	0.428	0.615	1.212	0.602	4.974	0.734	0.921
*p*-value	0.513	0.433	0.271	0.438	0.026	0.392	0.337
**Age**							
18–45	3.66 ± 0.77	3.69 ± 0.88	3.88 ± 0.89	3.99 ± 0.74	2.39 ± 0.86	3.73 ± 1.09	2.86 ± 1.11
46–69	3.73 ± 0.77	3.60 ± 0.92	4.01 ± 0.87	4.08 ± 0.76	2.32 ± 0.88	3.79 ± 1.01	2.75 ± 1.22
70 or above	3.64 ± 0.77	3.37 ± 1.00	3.91 ± 0.81	4.07 ± 0.74	2.27 ± 0.88	3.66 ± 1.01	2.53 ± 1.35
*F*-value	1.339	7.113	1.652	0.495	0.693	1.555	3.868
*p*-value	0.263	0.001	0.192	0.610	0.500	0.212	0.021
**Personal Annual income (Yuan)**							
≤ 5,000	3.66 ± 0.77	3.51 ± 0.98	3.87 ± 0.86	4.11 ± 0.68	2.27 ± 0.83	3.72 ± 0.99	2.55 ± 1.26
5,001–20,000	3.69 ± 0.79	3.47 ± 0.96	4.02 ± 0.88	4.11 ± 0.79	2.34 ± 0.90	3.64 ± 1.09	2.53 ± 1.32
≥20,001	3.71 ± 0.75	3.53 ± 0.93	4.03 ± 0.77	3.97 ± 0.80	2.31 ± 0.93	3.83 ± 0.96	2.97 ± 1.21
*F*-value	0.292	0.231	3.601	3.036	0.388	2.148	9.235
*p*-value	0.747	0.794	0.028	0.049	0.678	0.117	0.000
**Education qualification**							
Primary school and below	3.48 ± 0.71	3.29 ± 0.90	3.80 ± 0.91	4.01 ± 0.74	2.26 ± 0.85	3.46 ± 1.03	2.30 ± 1.30
Junior high school	3.66 ± 0.79	3.49 ± 1.01	3.99 ± 0.80	4.06 ± 0.76	2.36 ± 0.90	3.83 ± 0.96	2.85 ± 1.20
High school or above	3.96 ± 0.74	3.80 ± 0.91	4.13 ± 0.77	4.17 ± 0.74	2.29 ± 0.89	3.97 ± 0.99	2.92 ± 1.23
*F*-value	27.821	19.509	11.118	3.226	0.852	19.801	20.820
*p*-value	0.000	0.000	0.000	0.040	0.427	0.000	0.000
**Moxibustion usage experience**							
Yes	4.09 ± 0.69	4.04 ± 0.76	4.12 ± 0.71	4.32 ± 0.61	2.21 ± 0.79	4.26 ± 0.74	3.57 ± 0.85
No	3.30 ± 0.63	3.00 ± 0.85	3.80 ± 0.93	3.84 ± 0.79	2.39 ± 0.95	3.23 ± 0.99	1.81 ± 1.00
*F*-value	1.439	1.439	32.801	13.340	26.782	44.184	25.455
*p*-value	0.231	0.000	0.000	0.000	0.000	0.000	0.000
**Chronic disease**							
Yes	3.78 ± 0.73	3.78 ± 1.00	3.98 ± 0.81	4.10 ± 0.71	2.31 ± 0.95	3.77 ± 1.03	2.71 ± 1.22
No	3.64 ± 0.78	3.64 ± 0.94	3.95 ± 0.86	4.06 ± 0.76	2.30 ± 0.85	3.71 ± 1.01	2.64 ± 1.30
*F*-value	0.662	1.091	0.391	0.122	4.686	0.311	4.134
*p*-value	0.416	0.297	0.532	0.727	0.031	0.577	0.042

PE, performance expectancy; EE, effort expectancy; SI, social influence; FC, facilitating condition; PR, perceived risk; BI, behavioral intention; UB, usage behavior.

### 4.3 Measurement model

Prior to the formal survey, a pre-test was conducted with a pilot sample (*n* = 50). Based on the pre-test results, we performed preliminary reliability and validity analyses and subsequently revised the questionnaire by removing items with low factor loadings (< 0.5) or cross-loadings (>0.4). Cronbach's α coefficient was used to measure the internal consistency of the questionnaire (43). Table 4 shows that Cronbach's α coefficients of all variables are above 0.7, indicating a high degree of reliability of the questionnaire. To test for common method bias, Harman's single factor test was used to analyze all items of the key variables. The first factor explained 38.39% of the variance, which was below the standard threshold of 40%, indicating that common-method bias is unlikely to confound our results. Validity refers to the extent to which a measurement tool accurately reflects the intended measurement target (44), exploratory factor analysis (EFA) and confirmatory factor analysis (CFA) are jointly applied for validation.

**Table 4:** Reliability and convergent validity.

**Construct**	**Item**	**Factor loading**	**Cronbach's α**	**CR**	**AVE**
Performance expectancy (PE)	PE1	0.740	0.845	0.940	0.612
	PE2	0.908			
	PE3	0.784			
Effort expectancy (EE)	EE1	0.803	0.891	0.945	0.634
	EE2	0.906			
	EE3	0.861			
Social influence (SI)	SI1	0.920	0.862	0.950	0.660
	SI2	0.963			
	SI3	0.830			
Facilitating condition (FC)	FC1	0.760	0.921	0.922	0.798
	FC2	0.874			
	FC3	0.759			
Perceived risk (PR)	PR1	0.842	0.911	0.948	0.647
	PR2	0.958			
	PR3	0.843			
Behavioral intention (BI)	BI1	0.897	0.909	0.947	0.644
	BI2	0.876			
	BI3	0.859			
Usage Behavior (UB)	UB1	0.921	0.941	0.952	0.667
	UB2	0.962			
	UB3	0.872			

CR, composite reliability; AVE, average variance extracted.

Before conducting EFA, we performed Kaiser–Meyer–Olkin (KMO) and Bartlett's tests to evaluate the suitability of the survey data for factor analysis. The KMO value was 0.887, and the Sig value of the sample data chi-square statistic was 0.000, indicating sufficient correlations for factor analysis. The EFA results revealed all items loaded significantly (0.740–0.963) on their respective factors, with no cross-loadings exceeding 0.40. This factor structure aligned with the theoretical framework and justified the item deletions from the pre-test (Table 4). Then, confirmatory factor analysis (CFA) was conducted to verify the model derived from EFA. As shown in Table 4, the composite reliability (CR) ranged from 0.922 to 0.952, and the average variance extracted (AVE) ranged from 0.612 to 0.798, satisfying convergent validity criteria (45, 46). In Table 5, the correlation coefficient between the latent variable and all other variables was less than the square root value of the AVE of the latent variable, indicating that all constructs in this study support the discriminant validity of the data.

**Table 5:** Correlation matrix and square root of the AVE.

**Construct**	**AVE**	**UB**	**BI**	**PR**	**FC**	**SI**	**EE**	**PE**
UB	0.667	**0.817**						
BI	0.644	0.616	**0.802**					
PR	0.647	−0.089	−0.396	**0.804**				
FC	0.653	0.340	0.414	−0.273	**0.893**			
SI	0.798	0.200	0.476	−0.302	0.330	**0.812**		
EE	0.634	0.417	0.513	−0.329	0.420	0.243	**0.796**	
PE	0.612	0.551	0.593	−0.270	0.566	0.308	0.639	**0.782**

AVE, average variance extracted.

The first values of each column (bold) are the square roots of AVE values, and other values are the correlations among constructs.

### 4.4 SEM analysis and hypothesis testing

After ensuring the reliability and validity, we test the constructed structural equation model. The model fit indices of the SEM were all within specifications (RMSEA = 0.079, GFI = 0.895, CFI = 0.932, IFI = 0.932, NFI = 0.920, TLI = 0.920) (47), indicating good model fit. The standardized path coefficients and their significance levels are presented in Table 6. All hypotheses were tested at an adjusted significance level of 0.00625 (Bonferroni correction). Since all *p*-values were below 0.001, every hypothesis remained significant. Performance expectancy (*r* = 0.603, *p* < 0.001), effort expectancy (*r* = 0.260, *p* < 0.001), social influence (*r* = 0.373, *p* < 0.001), perceived risk (*r* = −0.162, *p* < 0.001) had direct effects on behavioral intention. Facilitating condition (*r* = 0.186, *p* < 0.01) and behavioral intention (*r* = 0.708, *p* < 0.001) had direct positive effects on usage behavior. And SI to PR (*r* = −0.334, *p* < 0.001), PR to PE (*r* = −0.194, *p* < 0.001) were significant. Thus H1, H2, H3, H4, H5, H6, H7, and H8 were supported.

**Table 6:** Standardized coefficients.

**Hypothesis**	**Path**	**Coefficient**	**SE**	**CR**	***p***	**Results**
H1	PE → BI	0.603	0.069	8.757	^***^	Supported
H2	EE → BI	0.260	0.047	5.567	^***^	Supported
H3	SI → PR	−0.334	0.041	−8.171	^***^	Supported
H4	SI → BI	0.373	0.044	8.502	^***^	Supported
H5	FC → UB	0.186	0.061	3.070	^**^	Supported
H6	PR → PE	−0.194	0.027	−7.192	^***^	Supported
H7	PR → BI	−0.162	0.039	−4.196	^***^	Supported
H8	BI → UB	0.708	0.045	15.913	^***^	Supported

SE, standard error; CR, critical ratio.

^***^p < 0.001, ^**^p < 0.01.

### 4.5 Multiple-group analysis

To test whether the proposed model had cross-group stability, this study conducted a multiple-group path analysis on demographic variables (i.e. income, usage experience and chronic disease prevalence). First, we grouped participants based on gender, usage experience and chronic disease prevalence. Then, we used parameter pairing to test individual paths. The effects of the moderating variables on different grouping situations were examined by structural modeling (Nested Model Comparison), which determines whether there is a moderating effect based on the critical ratios of the differences in path coefficients for the different cohorts. The critical ratio of 1.96 is used as a test standard (48).

In the experience grouping, we found that moxibustion usage experience plays a role in the path from FC to UB, PR to PE and BI to UI, with critical ratios between the parameters of −1.997, 2.298, and −2.246. In the Chronic disease grouping, we found that Chronic disease prevalence plays a role in the path from FC to UB, with critical ratios between the parameters of 2.286. Please refer to Table 7 for specific results.

**Table 7:** Multiple-group analysis.

**Hypothesis**	**Income**		**CRDP**	**Experience**		**CRDP**	**Chronic disease**		**CRDP**
	**Low-income**	**High-income**		**Yes**	**No**		**Yes**	**No**	
PE → BI	0.492^***^	0.703^***^	1.589	0.390^***^	0.437^***^	0.350	0.416^**^	0.654^***^	1.433
EE → BI	0.231^***^	0.289^***^	0.596	0.082	0.094	0.123	0.263^**^	0.269^***^	0.054
SI → PR	−0.319^***^	−0.353^***^	−0.430	−0.392^***^	−0.284^***^	1.299	−0.420^***^	−0.307^***^	1.169
SI → BI	0.437^***^	0.327^***^	−1.308	0.297^***^	0.427^***^	1.599	0.433^***^	0.347^***^	−0.822
FC → UB	0.190	0.190^*^	0.001	0.190^*^	−0.019	−1.997	−0.068	0.260^***^	2.286
PR → PE	−0.166^***^	−0.353^***^	−0.856	−0.223^***^	−0.108^***^	2.298	−0.179^***^	−0.204^***^	−0.466
PR → BI	−0.190^**^	−0.135^**^	0.714	−0.233^***^	−0.186^***^	0.654	−0.171^*^	−0.157^***^	0.162
BI → UB	0.699^***^	0.715^***^	0.192	0.493^***^	0.315^***^	−2.246	0.644^***^	0.736^***^	0.963

CRDP, critical ratio difference between parameters.

^***^p < 0.001, ^**^p < 0.01,^*^p < 0.05.

**Figure 2:**
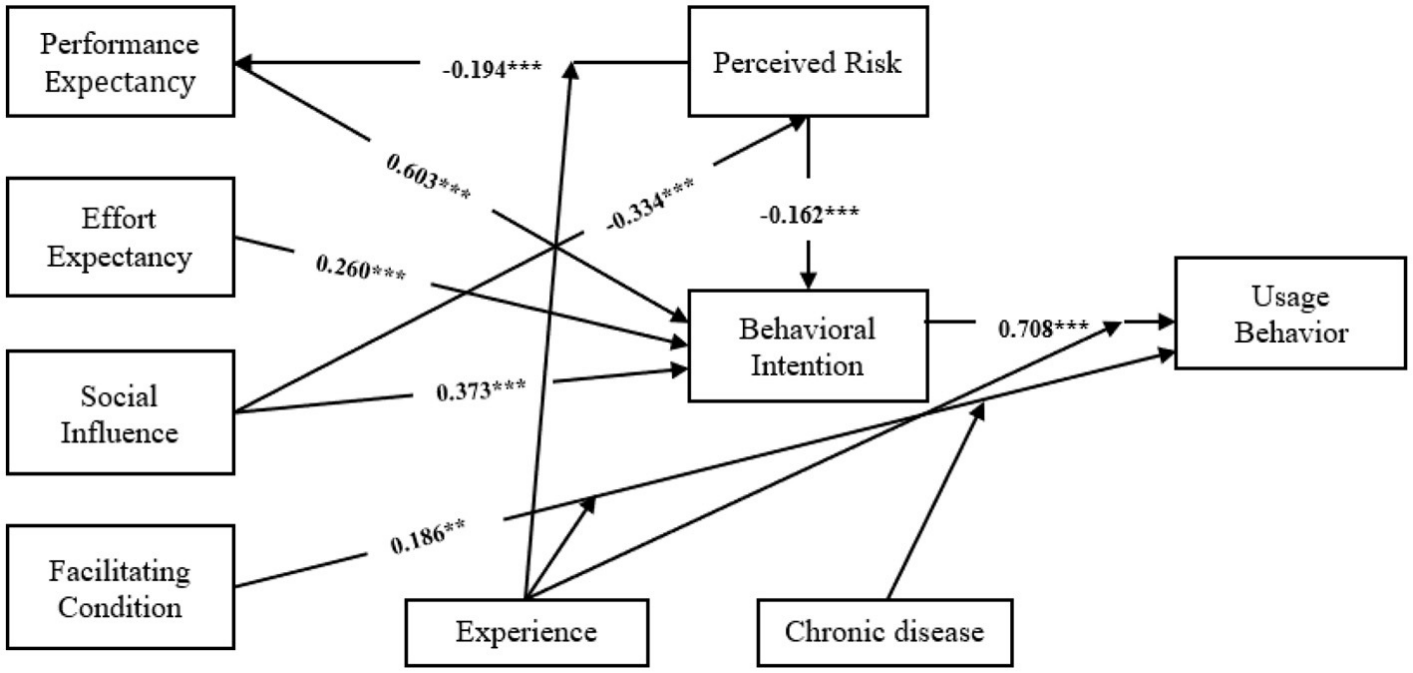
Path diagram of moxibustion application. ^**^p < 0.01, ^***^p < 0.001.

In summary, behavioral intention was influenced by performance expectancy, effort expectancy, social influence and perceived risk, in descending order of importance: PE > SI > EE> PR. Meanwhile, UB was influenced by FC and BI, with BI having the largest influence effect. In multi-group analysis, usage experience and chronic disease play a moderating role in the pathways (Figure 2).

## 5 Discussions

The current study aimed to investigate the factors influencing residents' behavioral intentions toward adopting moxibustion. Based on previous studies, we adapted and extended the traditional UTAUT model by incorporating characteristics specific to moxibustion and other appropriate TCM techniques, thereby introducing the measurement dimension of perceived risk factors. The main findings of the study are summarized as follows.

First, descriptive statistics indicated that the PR dimension received the lowest scores, suggesting that residents exhibit limited concern regarding the potential risks associated with moxibustion use. Conversely, this may reflect the insufficient dissemination of information about moxibustion at the current stage (49). Therefore, enhancing public education and awareness regarding moxibustion and other TCM healthcare techniques could effectively mitigate potential negative outcomes associated with their use.

One-way analysis of variance revealed statistically significant differences in usage behaviors among residents with varying ages, income levels, educational backgrounds, moxibustion usage experiences, and prevalence of chronic diseases. Specifically, residents aged 18–45 demonstrated significantly higher usage behaviors compared to other age groups. As a TCM technique, moxibustion's application is influenced by residents' health status and technical proficiency, with its operation becoming more challenging as age increases (50). Additionally, higher income levels were associated with increased average scores, aligning with previous research findings (51). Indicating that a certain amount of financial support is needed to carry out moxibustion treatments. Higher-income groups tend to prioritize their healthcare and are more inclined to utilize moxibustion to enhance their health status. Regarding chronic disease prevalence, residents with chronic conditions who had previously used moxibustion exhibited significantly higher mean scores. This finding is consistent with previous research (52). Previous studies have indicated that individuals with chronic illnesses possess a heightened awareness of their health conditions and a greater demand for healthcare technologies, facilitating their adoption and utilization of such services (53).

Second, each path in the structural equation model was significant. Among the factors influencing BI, PE showed the highest path coefficient. This finding is consistent with existing studies (44, 54), indicating that residents' behavioral intention is primarily influenced by their perception of the technology's usefulness. EE have a positive impact on residents' intention to use moxibustion, this finding confirms previous research (32, 55). This suggests that when residents perceive moxibustion as easier to use, their behavioral intentions to adopt the technique are significantly and positively influenced. SI significantly affects both residents' intentions to use moxibustion and their perceived risks associated with its use. This finding differs from previous findings (56). As an important source of health information, physicians, and social networks significantly influence residents' trust in healthcare technology. This finding has practical implications, suggesting that concerns regarding moxibustion risks can be mitigated through information sessions and sensitization of medical personnel. PR have a negative effect on residents' intention to use moxibustion, this finding validates similar findings in the field of TCM technology (37, 57), and highlighting safety as a critical consideration for potential users. In addition to concerns about the inherent safety and efficacy of the technology, these risks also emerge from the insufficient or ambiguous dissemination of information. From a practical perspective, continuous improvement in both the quality of the technology and service standards, along with targeted educational interventions, can play an essential role in reducing perceived risks and thereby encouraging broader adoption. FC and BI have a significant impact on residents' usage behavior. Psychological theories posit that behavioral intentions govern actions, thereby explaining the influence of willingness on actual usage behavior. This view has been confirmed in related studies (39, 58). When residents perceive moxibustion technology as user-friendly and receive comprehensive assistance from experts, their likelihood of adopting the technology increases significantly (35).

Third, the multi-group analysis indicated that moxibustion usage experience plays a moderating role in the path of FC to BI and PR to BI. Residents with prior moxibustion experience demonstrate a deeper understanding of the technique and perceive it as more user-friendly, thereby strengthening the positive impact of FC on BI. Concurrently, their prior experience allows them to assess potential risks more accurately, mitigating the negative impact of PR on BI. Furthermore, the multi-group analysis revealed significant differences in the path from FC to UB between residents with and without chronic diseases. Specifically, FC positively influenced UB among residents with chronic conditions, suggesting that the availability of supportive infrastructure enhances their engagement with moxibustion practices. Conversely, for these individuals without chronic diseases, increased facilitating conditions might not translate to higher usage, possibly due to differing motivations or perceptions of necessity. This divergence underscores the importance of tailoring moxibustion promotion strategies to address the distinct needs and perceptions of different user groups.

## 6 Limitation and future research

In this study, we expanded the traditional UTAUT measurement model. Based on the characteristics of moxibustion and other TCM healthcare techniques, we added the perceived risk factor and the study confirmed that it would have a direct impact on the willingness to use moxibustion. Additionally, this study explores the factors influencing moxibustion use from an individual's perspective, providing valuable insights for TCM technical service providers, planners, and policymakers. However, this study also has some limitations. First, this study was conducted exclusively in Shandong province. Given the variations in residents' levels of TCM health literacy across different regions, the representativeness of research samples and the generalizability of the findings are somewhat limited. Future studies should be conducted on populations from diverse regions and social backgrounds in China. Second, although the UTAUT model was expanded in this study, certain items were removed in the pilot testing phase on the reliability and validity analysis of the questionnaire. Consequently, the remaining variables may not completely capture the essence of the measurement dimension. Future studies should aim to refine the survey scale to ensure a more comprehensive assessment of the constructs involved. Last, the study relies solely on self-reported data from a cross-sectional survey, which may introduce recall and social desirability biases, and limits the ability to infer causality. Future research should incorporate objective measures and longitudinal designs to enhance validity.

## 7 Conclusions

This study applied the UTAUT framework to develop and empirically test a model of residents' acceptance of moxibustion. Aligning with China's Healthy China initiative and the strategic aim of advancing high-quality development in the Chinese medicine industry. Based on the constructed research model, we explored the factors that influence residents' behavioral intention to adopt moxibustion across five dimensions: Performance Expectancy, Effort Expectancy, Social Influence, Facilitating Conditions, and Perceived Risk. Our research findings indicate that enhancing expectations of moxibustion's performance and ease of use, cultivating a supportive social environment, ensuring adequate infrastructural and logistical support, and reducing perceived risks are all critical strategies for promoting residents' engagement and sustained utilization of moxibustion services.

## Data Availability

The raw data supporting the conclusions of this article will be made available, upon reasonable request. Requests to access the datasets should be directed to WY, yinwq1969@126.com.
